# Treatment of Chronic Trigeminal Neuralgia

**Published:** 1879-02-20

**Authors:** 


### *TREATMENT OF CHRONIC TRIGEMINAL NEURALGIA.*

Dr. E. C. Seguin reported three cases of chronic trigeminal neuralgia which were cured by medicinal treatment.

Dr. Grey inquired whether any member had tried the galvanic current in the treatment of trigeminal neuralgia. He referred to a case reported by Niemeyer, in which cure was effected by the use of the galvanic current after all other known means had failed; and also to a case under his own care, in which violent neuralgia of the thigh was relieved by the same means and under like circumstances.

Dr. Kinnicutt remarked that he had employed the galvanic current in the treatment of acute cases of trigeminal neuralgia, and that relief had been afforded, but it was only temporary.

Dr. Seguin remarked that the galvanic current was a measure which should be used more than it was, but there were practical difficulties which to a very great extent rendered it unavailable. It was rare that the physician could spare the necessary time, or the patient meet the necessary expense attending that method of treatment.

Dr. Spitzka remarked that heretofore, in cases of trigeminal neuralgia, when he had not been able to discover indications for treatment arising from reflex causes, or central disease, or constitutional phases, he had told his patients that he could do nothing for them whatever; but, from the results obtained in the cases reported, he should be encouraged to adopt the plan recommended by Dr. Seguin. He then asked the following questions :

1. What were the indications for the use of aconitia in the treatment of cases of trigeminal neuralgia?

2. Why was the term epileptiform used in connection with one of the cases reported?

3. Should not the diplopia, the unequal pupils, the loss of memory in one case, be regarded as symptoms pointing to some general central disease?

With reference to his own experience in the use of aconitia in other painful affections, pushing the remedy until its physiological effects were produced had seemed to be a rather dangerous procedure in some patients.

In one case he had pushed the remedy so that it simply produced a slight tingling, and had not strong measures been resorted to the case would have terminated fatally.

Dr. Seguin in reply stated:

1. That he knew of no special condition which indicated the use of aconitia.

2. He uses the term epileptiform because it best described the suddenness with which the attacks came on.

3. He did not believe the patient referred to had any central disease, for he had tested him in every direction with the view to such discovery, and had found nothing.

With reference to impairment of memory, it could be reasonably expected that the memory of a man who for ten years had suffered untold agony, might be affected.

The contracted pupil was upon the same side with the pain, and was due probably to simple irritation of the trigeminus.

The diplopia was present only while the patient was taking medicine.

Dr. Kinnicutt remarked that he was induced to use ergot in the treatment of one case of trigeminal neuralgia of eight years' standing, because the nitrite of amyl always produced bad effects. The results obtained by the use of the ergot, given in doses of one drachm and a half of the aqueous extract in the course of twenty-four hours, had been favorable.

Dr. G. M. Beard remarked that the conclusions arrived at by the author of the paper harmonized with his own convictions regarding the use of powerful remedies and pushing them until marked physiological effects were produced. The idea was old, it was true, and there had also been a reaction against it which had been carried too far. The principle was illustrated in the treatment of syphilis and other diseases in which the best results were obtained by producing the physiological effects of powerful drugs.

With regard to the use of electricity in the treatment of the disease under consideration, judging from his own experience, the prognosis certainly was very bad, while in the treatment of ordinary trigeminal neuralgia the prognosis was very good. He had no recollection of ever having cured a case of epileptiform neuralgia by the use of electricity. He had not been able to confirm the German case referred to, and regarded the result obtained there as an exception, the like of which might not be seen in a thousand years to come. Such was not the fact, however, with reference to ordinary trigeminal neuralgia.

Dr. Beard thought all would agree that neuralgia was only a symptom, and that when twenty cases were treated, we might in reality be treating twenty different diseases. Therefore it was not safe to generalize with reference to the action of any special remedy.

With reference to the use of gelseminum, his custom was to begin with doses of from three to five drops, and gradually increase. If no idiosyncrasy was discovered, he ceased to fear the ill effects of the remedy, and did not hesitate to push its administration to full doses.

Dr. Seguin wished to record his favorable experience in the use of phosphorus in the treatment of ordinary trigeminal neuralgia. He had obtained more satisfactory results from its use than from the use of gelseminum.

Dr. Grey remarked that he had been made aware of the danger of pushing powerful remedies until they produce their full physiological effects, and referred to cases of chorea, in which he had seen dangerous symptoms produced by the use of arsenic.

Dr. Shaw shared in the opinion that pushing remedies until they produced full physiological effects was the true method for obtaining the most satisfactory results in the treatment of many diseases.

Dr. McBride directed attention to the importance of determining that no cardiac disease was present in cases which were to be treated by the use of aconitia.—*Neurological Society Reports.—N. Y. Med. Record.*

